# Direct Oral Anticoagulants (DOACs) for Therapeutic Targeting of Thrombin, a Key Mediator of Cerebrovascular and Neuronal Dysfunction in Alzheimer’s Disease

**DOI:** 10.3390/biomedicines10081890

**Published:** 2022-08-04

**Authors:** Klaus Grossmann

**Affiliations:** Center for Plant Molecular Biology (ZMBP), University of Tübingen, 72076 Tübingen, Germany; klaus.grossmann@uni-tuebingen.de

**Keywords:** Alzheimer´s disease, blood–brain barrier dysfunction, cerebral amyloid angiopathy, inflammation, vascular dysfunction, amyloid-beta, tau, thrombin, fibrin, direct oral anticoagulants

## Abstract

Although preclinical research and observer studies on patients with atrial fibrillation concluded that direct oral anticoagulants (DOACs) can protect against dementia like Alzheimer’s disease (AD), clinical investigation towards therapeutical approval is still pending. DOACs target pathological thrombin, which is, like toxic tau and amyloid-ß proteins (Aß), an early hallmark of AD. Especially in hippocampal and neocortical areas, the release of parenchymal Aß into the blood induces thrombin and proinflammatory bradykinin synthesis by activating factor XII of the contact system. Thrombin promotes platelet aggregation and catalyzes conversion of fibrinogen to fibrin, leading to degradation-resistant, Aß-containing fibrin clots. Together with oligomeric Aß, these clots trigger vessel constriction and cerebral amyloid angiopathy (CAA) with vessel occlusion and hemorrhages, leading to vascular and blood–brain barrier (BBB) dysfunction. As consequences, brain blood flow, perfusion, and supply with oxygen (hypoxia) and nutrients decrease. In parenchymal tissue, hypoxia stimulates Aß synthesis, leading to Aß accumulation, which is further enhanced by BBB-impaired perivascular Aß clearance. Aß trigger neuronal damage and promote tau pathologies. BBB dysfunction enables thrombin and fibrin(ogen) to migrate into parenchymal tissue and to activate glial cells. Inflammation and continued Aß production are the results. Synapses and neurons die, and cognitive abilities are lost. DOACs block thrombin by inhibiting its activity (dabigatran) or production (FXa-inhibitors, e.g., apixaban, rivaroxaban). Therefore, DOAC use could preserve vascular integrity and brain perfusion and, thereby, could counteract vascular-driven neuronal and cognitive decline in AD. A conception for clinical investigation is presented, focused on DOAC treatment of patients with diagnosed AD in early-stage and low risk of major bleeding.

## 1. Introduction

Alzheimer’s disease (AD) is the result of a complex syndrome from neurodegenerative, vascular, and hemostatic alterations, mainly in neocortical and hippocampal brain areas. AD pathogenesis causes memory, cognition, behavioral and motor abilities, and, ultimately, the known personality of a human to gradually vanish. Worldwide, more than 40 million people, including 1 million alone in Germany, suffer from this disease, with a rising prevalence due to the global increase in the most affected aging population [1,2]. Of these patients, lower than 10% develop symptoms due to hereditary and genetic disposition long before the age of 65 in the course of early-onset AD [3].

Current drugs for AD treatment are only able to alleviate disease symptoms and delay memory loss by a few months without effect on the main pathological processes of the disease [4]. In the first mild or moderate stages, cholinesterase inhibitors, such as donepezil, galantamine, and rivastigmine, have been the standard AD care for more than 25 years [4]. In later and more severe stages, cholinesterase inhibitors are often combined with glutamate antagonists such as memantine [4]. Changes in lifestyle and diet and avoiding of cardiovascular risk factors are also considered to play a preventive and delaying role [5]. What is still missing today despite decades of intensive research and investment are novel drugs already available for clinical utility, or visible in the research pipeline [6]. Disease-modifying agents are particularly desirable for effectively combating this disease due to targeting its causes. Of lesser importance are symptomatic agents, which affect only the symptoms of the disease [2,4,7]. Although the pathogenesis of AD is still not fully understood, one of the primary triggering mechanisms is ascribed to the accumulation of toxic amyloid-ß-proteins (Aß) in the diseased brain. Recently, the U.S. Food and Drug Administration (FDA) has approved a new disease-modifying therapy using the anti-Aß-antibody aducanumab [2,4,7]. Aducanumab targets Aß and clears these peptides, and is thereby intended to combat this causative factor. However, approval of aducanumab for the treatment of mild AD has sparked a controversial discussion, whether this antibody can be sufficiently effective to slow memory loss and cognitive decline, as indicated by the submitted clinical trials [2,4,7]. On the other hand, a certain spirit of optimism is recognizable in pharmaceutical research and in public. The opportunity is now seen again to identify medication that can improve symptoms more significantly, can slow AD progression, can delay the onset, or can even prevent this feared brain disease [2,3,4,8].

Nevertheless, the process of pharmaceutical drug development is both time- and cost-consuming. Therefore, an alternative therapeutic approach is becoming more and more common that relies on repurposing of known drugs which are approved for other indications but which could also impact AD, possibly through on-target or off-target mechanisms [8]. These drugs have the advantage that their safety profiles, pharmacokinetics, formulations, doses, and manufacturing processes are known. Therefore, drug development costs and time for progression in the pipeline could be substantially reduced [8]. In this context, anticoagulants could be promising therapeutic candidates for targeting hemostatic and cerebrovascular dysfunctions and associated neurodegenerative processes in AD [9,10,11]. After first clinical evaluation more than 50 years ago, the renaissance of an anticoagulative therapy has been inspired by recent research that indicates that cerebrovascular damage and dysregulated intrinsic coagulation, as well as risk factors for cardiovascular disease, play important roles in AD pathogenesis [9,12,13]. Recently, quantitative transcriptome profiling of major vascular and perivascular cell types from hippocampal and cortical brain samples of individuals with AD has also indicated dysregulation of blood flow [14]. Interestingly, the gene expression patterns linked 30 of the top 45 genes for AD risk to vasculature [14]. Particularly, it turned out that accumulations of toxic Aß, along with excessive thrombin and fibrin(ogen) accumulations, are causally involved in cerebrovascular and blood–brain barrier (BBB) dysfunction. These changes might be mechanistically treated by anticoagulants through targeting its causes [9,10,11,15]. This article presents a comprehensive review on latest studies, showing that anticoagulants, especially of the DOAC-type, can be a promising option for a successful medication of vascular-conditioned, cognitive impairment in AD.

## 2. Hemostasis, Thrombosis, and Antithrombotic Medication

### 2.1. Blood Coagulation and Fibrinolysis

In hemostasis, the vascular wall, platelets, and the coagulation and fibrinolysis system in the blood interact functionally together in order to (i) quickly seal injured vessels with a clot of blood, a thrombus, and thus stopping bleeding; (ii) limit thrombus formation to the area of vascular injury; and (iii) remove the thrombus during wound healing [16]. The formation of a thrombus involves platelet activation through endothelial cells of the damaged blood vessel and the initiation of multiple, closely linked and regulated biochemical processes in the plasmatic coagulation system via extrinsic and intrinsic pathways [16]. At the common connection of both pathways, activation of the key enzyme of blood clotting, the serine protease thrombin (factor IIa), takes place by the prothrombinase complex. Thrombin cleaves fibrinogen, a soluble protein, which then polymerizes to insoluble fibrin protofibrils. Fibrinogen normally circulates in the blood in huge quantities, released particularly by the liver. Fibrin protofibrils form a stable construct of cross-linked fibrin strands with integrated erythrocytes and platelets, known as a blood clot or thrombus. In addition, fibrin(ogen) can trigger inflammatory processes in neurodegeneration [17,18].

The structure of thrombi is determined particularly by the flow velocity of the blood and the resulting shear forces and by their content of platelets, which is high in arterial vessels and low in venous ones. Thrombin and fibrin(ogen) also promote blood clotting by forming bridges between and activating platelets. The production of thrombin from the protein precursor prothrombin is controlled by an upstream series of processes. These are partially vitamin K dependent and contain multiple tissue and coagulation factors, such as factor Xa (FXa), a key enzyme of the prothrombinase complex [16]. Moreover, the proteolytic enzymes of the plasmatic coagulation system in the blood are opposed to differently acting specific inhibitors, such as antithrombin, which particularly interacts with thrombin and FXa [16].

Parallel to fibrin formation, the plasmatic cascade of fibrinolysis is initiated by the release of tissue plasminogen activator (t-PA) particularly from endothelial cells [16]. The function of fibrinolysis is to dissolve intravascular fibrin clots and stop thrombus growth, as well as to remove the thrombus during wound healing. t-PA activates fibrin-bound plasminogen, which is then proteolytically cleaved to form the serin protease plasmin. Plasmin catalyzes the degradation of insoluble fibrin into soluble fibrin fission products and thereby dissolves fibrin clots [16]. Platelet functioning, as well as the process cascades of coagulation and fibrinolysis, are subjected to intrinsic control mechanisms, which prevent excessive thrombus formation [16]. If these hemostatic processes are disturbed, e.g., in hemophilia by hereditary-conditioned deficiency of a coagulation factor, the risk of bleeding is increasing. On the other hand, the risk of severe thrombus formation rises with appearance of major wounds in the course of surgeries and injuries, as well as in conditions of slow blood flow, e.g., in atherosclerotic vascular disease, heart arrhythmias, such as atrial fibrillation (AF), and restricted physical exercise [16]. Stationary thrombi can occlude blood vessels in the process of arterial and venous thrombosis. In addition, an attached thrombus on the vessel wall can break loose and migrates through the bloodstream to organs as a thrombo-embolus, which can cause, e.g., heart attack, pulmonary embolism, or brain infarction [16].

### 2.2. Antithrombotic Therapy

#### 2.2.1. Drug Portfolio

Overall, thrombotic events are a major complication of cardiovascular diseases, with fatal consequences if not appropriately treated by antithrombotic medication [16]. The available portfolio comprises three categories of drugs: 1. platelet aggregation inhibitors (PAIs), which prevent blood clots in long-term management of arterial thrombosis by blocking cyclooxygenase (COX) or a platelet surface receptor; 2. fibrinolytics, which permit lysis of an already formed thrombus and thereby vessel reperfusion by converting plasminogen to fibrin-dissolving plasmin; and 3. anticoagulants [16].

Over the last 80 years, anticoagulants have been used to prevent fatal thromboembolic events by blocking the coagulation cascade for forming fibrin clots. The first anticoagulative drug for the treatment of patients with cardiovascular disease was the orally active coumarin derivative dicumarol, which was launched into clinical practice in 1941 [19]. This plant substance, originally isolated from spoiled sweet clover hay, acts as a vitamin K antagonist (VKA). It indirectly blocks multiple vitamin K dependent processes in the coagulation cascade, leading to an anticoagulant effect. Dicumarol and derivatives competitively inhibit microsomal vitamin K epoxide reductase, which converts inactive vitamin K 2,3-epoxide to reduced, active vitamin K. Vitamin K is needed for the carboxylation of glutamate residues in the synthesis of the coagulation factors II, VII, IX, and X and proteins C and S [16,19,20]. Later, further VKA-type anticoagulants, such as warfarin (Coumadin^®^), phenprocoumon (Marcumar^®^, Falithrom^®^), and acenocoumarol (Sintrom^®^), were introduced into pharmaceutical market. They found particular attention and momentum for therapy of cardiovascular diseases after treatment of US President Eisenhower´s heart attack in 1955 [19]. However, VKA-type anticoagulants have serious disadvantages, such as bleeding complications and drug–food interactions, which demand close monitoring of drug levels in the blood [16,20].

Nowadays, a palette of anticoagulants with different dosages, forms, and mechanisms of action is available, which can affect the plasmatic coagulation cascade indirectly, as in the case of the long-known, oral-active VKAs and of parenteral heparins (e.g., enoxaparin, a low molecular weight glycosaminoglycan form of heparin), heparinoid danaparoid sodium, and fondaparinux [16]. Heparins inactivate thrombin and FXa through an antithrombin-dependent mechanism [16]. On the other hand, both components of the coagulation cascade can also be inhibited directly, independent of antithrombin. Thus, thrombin is directly blocked, e.g., by parenteral hirudin, bivalirudin, argatroban. Likewise, orally active dabigatran binds to and thus directly inhibits both soluble and fibrin-bound thrombin [16]. Dabigatran is administered as the prodrug form dabigatran etexilate (Pradaxa^®^). On the other hand, for direct FXa inhibition, oral-active drugs, including apixaban (Eliquis^®^), betrixaban (Bevyxxa^®^), edoxaban (Lixiana^®^), and rivaroxaban (Xarelto^®^), are available. They bind, selectively and reversibly, to the active site of activated factor FX (FXa) in an antithrombin-independent manner [16]. Both the direct thrombin-inhibitor dabigatran and the FXa-inhibitors represent the newest category of anticoagulants, summarized as direct oral anticoagulants (DOACs). These specific-acting DOACs, which were launched into the pharmaceutical market in the last 15 years, are characterized by easy dosing regiments, no food–drug interactions, and no need for constant patient monitoring, as well as by a reduced risk of dangerous intracranial bleeding [9,11,16,20]. DOACs are approved for a broad spectrum of cardiovascular indications, in order to prevent stroke and systemic embolism in patients, who exhibit, e.g., nonvalvular atrial fibrillation (AF) or increased cardiovascular risk factors, such as heart failure and high blood pressure. Likewise, DOAC therapy and prophylaxis in deep vein thrombosis and pulmonary embolism is indicated [16,20]. Today, DOACs are prescribed for antithrombotic treatments alone in Germany in approximately 2 million patients, mostly over 70 years of age [16,21]. In contrast, the number of prescriptions of VKA-type anticoagulants decreased in the last years and currently amounts to approximately 1 million patients in Germany [21].

#### 2.2.2. Fields of Indications

Currently, anticoagulants are used as short- and long-term options to prevent and to treat blood clots that may occur in blood vessels and cause stroke and thromboembolism events [16]. Short-term treatment is required in acute venous thrombosis but also for prophylaxis of thrombotic events in risk situations, such as surgeries. Long-term to permanent anticoagulation is ordained for the prevention of thromboembolic complications in patients who show heart arrhythmias (e.g., AF) and cardiovascular and thrombophilic risk factors or are equipped with a mechanical heart valve substitute [16]. Particularly, patients with AF have a five-fold increased incidence of stroke, which is associated with a high risk of disability and death [16,19]. The benefit of the antithrombotic prophylaxis and therapy is high and drastically reduces the risk of myocardial infarction, ischemic stroke, and mortality in vulnerable persons. Nevertheless, a certain risk of intracranial and gastrointestinal hemorrhage must be taken into account when treatment is prescribed [16]. Recently, a completely new aspect for application of anticoagulants has come into focus, which is based on new findings about the impact of vascular and hemostatic dysfunction in AD pathogenesis. Therefore, medical repositioning of anticoagulants towards therapeutic and prophylactic use for AD is in discussion, and clinical studies for approval are recommended [8,9,10,11,15].

## 3. Toxic Proteins and Chronic Inflammation in AD

Alois Alzheimer, a German psychiatrist and neuropathologist, was the first to describe dementia, which was later named “Alzheimer´s disease” after him, in 1906 at a conference of the university of Tübingen. He attributed symptoms of mental confusion and forgetfulness, which he had observed in a patient, to post-mortem protein deposits diagnosed in her brain tissue [22]. Indeed, recent findings suggest that neurofibrillary tangles, formed by tau proteins, and extracellular deposits of Aß that were later named senile plaques are early brain hallmarks for AD. Misfolded, toxic Aß have been found to aggregate and to form deposits of oligomers and fibrillar Aß plaques in the brain parenchyma, associated with the progressive loss of neurons, synapses, dendrites, myelin, and brain tissue [23,24,25,26,27,28,29,30,31]. Intriguingly, the first protein characterization of Aß was carried out in cerebrovascular amyloid material, isolated from human AD and Down´s syndrome brains in the 1980s [23,24].

### 3.1. Generation and Occurrence of Aß

The accumulation of misfolded, toxic oligomers of Aß in the AD brain is thought to be the result of pathological dyshomeostasis between progressive Aß production and failure of their clearance. In the “amyloid hypothesis of AD”, Aß are seen in the center of the key factors that initiate disease pathogenesis with its cascade of events, which include the development of tau neurofibrillary tangles, oxidative stress, and inflammatory and neurodegenerative processes [23,24,25,26,27,28,29,30,31]. Currently, this hypothesis provides the most important starting point for the search of novel drugs that can possibly slow, stop, cure, or prevent the disease [4,24,28,30]. In the amyloidogenic pathway for the generation of toxic Aß, Aß are excised from a single-domain membrane protein, the amyloid-ß precursor protein (AßPP), which is cleaved by sequentially acting transmembrane proteases, the ß- and γ-secretase [23,24,25,26,27,28,29,30,31]. Particularly, mutations in the Aß region of the *AßPP* precursor gene and in the presenilin subunits of the γ-secretase lead to aggressive Aß forms. These toxic Aß species are associated with early onset familial AD and vary in size, including Aß isoforms of 40 (Aß40) and 42 (Aß42) amino acids as the most abundant. A third AßPP splitting enzyme, the α-secretase, is involved in a nonamyloidogenic pathway, which does not contribute to the production of amyloid plaques. AßPP is mainly embedded in the plasma membrane of different types of neurons and glial cells. After secretase cleavage of AßPP, extracellular, soluble fragments of different length are produced, which include secreted AßPP fragment and Aß isoforms. Among these isoforms, oligomeric Aß40 of the shorter subtype is the predominant one, while Aß42 is more neurotoxic and aggregates faster than Aß40. Aß42 is the major species observed in plaques. Aß42 oligomers are also able to enter cells via endocytosis and to cause lysosomal fusion dysfunction. This effect can lead to increasing excretion of modified Aß into the extracellular space and can reduce Aß elimination by microglia phagocytosis [24,25,26,27,28,29,30,31].

At physiological level in the healthy brain, soluble Aß (predominantly Aß40, Aß42) and AßPP fragments are thought to be required for (i) synaptic functioning and neuronal survival [4]; (ii) repairing leaks in the vascular interface to the brain, the blood–brain barrier (BBB); and (iii) for the defense against pathogen infections [28]. In conditions of excessive Aß generation in the diseased AD brain, toxic Aß are secreted into the extracellular space, and accumulate initially as self-aggregating Aß monomers into soluble dimers, fibrillar oligomers, and polymers (protofibrils). Then, they are deposited as insoluble fibrils and fibrillar Aß plaques between neurons. Especially, fibrillar Aß seem to be a reservoir and source of toxicity to neuronal cells because the fibril surface can catalyze the conversion of Aß monomers into toxic oligomeric species [32]. In addition, multiple distinct fibril structures, called fibril polymorphs, are generated by the different Aß isoforms, which may differ in their neurotoxic potential [33]. Recently, cryo-electron microscopy structures of Aß42 filaments from the human brain indicate that two types of filaments exist, where type I is mostly found in persons with sporadic AD and type II in persons with familial AD [31]. Accumulation of the most neurotoxic and pathogenic Aß40 and Aß42 is accompanied by the interaction of their oligomeric isoforms with neuronal plasma membranes, synapses, microglia, and astrocytes. These interactions are seen as an important reason of their toxicity, together with their ability to spread spatiotemporally throughout the brain via seeded aggregation of a prion-like mechanism. Three major types of Aß deposits are widespread in the AD brain, parenchymal diffuse and focal deposits and vascular deposits [31]. Diffuse deposits, which consist of loosely packed Aß filaments, are detected in various brain areas, including the entorhinal cortex, presubiculum, striatum, brainstem, cerebellum, and subpial area. Focal deposits of dense core plaques consist of a spherical core of closely packed Aß, surrounded by more loosely packed filaments. Dense core plaques are observed mainly in the hippocampus and cerebral cortex. Particularly, filamentous Aß42 are found in the parenchymal diffuse plaques and the loosely packed filaments of dense core plaques. On the other hand, both Aß40 and Aß42 form parenchymal plaque cores, as well as deposits in vessel walls, with Aß40 subtype prevailing [31].

Parenchymal Aß are transported within the brain´s interstitial fluid (ISF) along the walls of blood vessels to the meningeal cerebrospinal fluid (CSF) and lymphatic vessels. The removal of parenchymal Aß is primarily from the ISF by transfer into the blood vessels across the BBB [34]. This process is called perivascular Aß clearance [34]. With increasing parenchymal Aß release into the blood, Aß oligomers (in particular subtype Aß40) deposit around and in the walls of leptomeningeal and cortical blood vessels. This cerebrovascular Aß cause a disease known as Aß-type cerebral amyloid angiopathy (CAA), which is associated with AD pathogenesis [5,27,34,35,36,37]. CAA affects vascular activity (vasoactivity) and functioning, which again also interferes with perivascular Aß clearance. Concomitantly, vascular Aß clearance is additionally impaired by a decrease in the diameter of meningeal lymphatic vessels for Aß drainage. This further amplifies Aß accumulation in the brain parenchyma.

### 3.2. Brain Locations and Pathogenic Action of Aß

The preferential areas in human AD brain, where vascular Aß deposits and parenchymal Aß dense core plaques are diagnosed, are the neocortex and hippocampus, which are key for higher-order cognition, behavior, and motor skills [31,38]. The neocortex is involved in brain functions, which include sensory perception, motor commands, cognition and spatial reasoning, social and emotional behavior, memory, as well as learning and language processes. The hippocampus acts as a switchboard between perception and memory [39]. Early in AD, these cerebral areas show the dysfunction and hyperactivity of neurons, which are accompanied by progressing synapse and neuron cell death, closely correlated with the severity of cognitive impairment [29,40].

#### 3.2.1. Evidence for Aß Causality

A series of recent findings strongly suggests that Aß are causally involved in AD pathogenesis. The main arguments for a key role of Aß in neurodegeneration are based on the following results. (i) High levels of soluble Aß dimers and oligomers have been found to affect normal transmission of neurons and induce neuronal hyperexcitability [4,29,41]. The mechanism behind this is that Aß block the reuptake of synaptically released glutamate in glutamatergic neurons, leading to neuronal hyperactivation and damage. This effect is additionally potentiated by Aß-inhibited glutamate uptake in astrocytes. (ii) In an AD mouse model, follicle-stimulating hormones (FSH) from the pituitary gland have been shown to act directly on hippocampal and cortical neurons, accelerating Aß and tau deposition, associated with cognitive impairment [42]. Blocking FSH action by an anti-FSHß antibody prevented AD effects through inhibiting the neuronal C/EBPß-δ-secretase pathway. This pathway leads to the cleavage of AßPP and tau, thus enhancing the formation of Aß and tau aggregates [42]. (iii) The major gene alterations, assigned to an increased risk of AD, have to do with the generation, aggregation, and clearance of Aß, as well as with associated microglial responses [3]. Assigned with familial AD are particularly genotypes, which exhibit multiplications of the *APP* gene encoding AßPP. This genotype is present, e.g., in individuals with Down´s syndrome (trisomy 21), indicating *APP* localization on chromosome 21 [3,23]. Likewise, mutations in the presenilin genes *PSEN1* and *PSEN2*, forming part of the γ-secretase complex, are genetic risk factors [3]. Relevant for AD risk are also mutations which increase the ratio of Aß42 to Aß40 or raise the concentration and assembly of Aß42 into filaments [31]. (vi) The use of Aß-targeting antibodies in AD mouse models as well as in patients was able to reduce both brain Aß load and cognitive impairment [30,43].

#### 3.2.2. Aß-Targeting Antibody Therapy

Passive immunotherapy with the human, monoclonal anti-Aß oligomers antibody aducanumab has shown potential to reduce brain Aß plaques and cognitive impairment in AD patients when treated early in the disease [2,7,30,43]. In 2021, aducanumab was approved by the FDA as the only disease-modifying medication up to now (marketed by Biogen as Aduhelm^®^), which potentially slows down AD progression [2,7]. However, one of the conditions for FDA approval was that a confirmatory trial, carried out by Biogen in the next nine years, ensures the clinical benefit of the antibody. Moreover, further anti-Aß antibodies, such as donanemab, gantenerumab, lecanemab, and solanezumab, which target Aß aggregation, fibril elongation, or plaque nucleation, are currently under clinical observation [2,7,30,44]. Indeed, studies have shown that these Aß-targeting antibodies are also able to lower amyloid load in cognitive-relevant brain regions, on average by up to 80% [30]. However, until now, the clinical benefit of these putative disease-modifying therapeutics has been rather modest, showing 20–40% deceleration of cognitive decline in 18-month trials [30]. In addition, anti-Aß antibodies can provoke unwanted side effects, such as brain swelling and microhemorrhages, which are mostly harmless, but which can be serious [2]. Moreover, Aß plaques are also found in the brain of non-demented, elderly persons. However, compared to these individuals, in AD patients, structurally different polymorphs of Aß fibrils are detected on average, and the quantity of Aß is greater, correlating with the severity of cognitive impairment [33]. Structural polymorphism of toxic protein fibrils, coupled with varied biological effect, have also been shown in other neurodegenerative diseases, such as spongiform encephalopathies, synucleopathies, and tauopathies [33].

### 3.3. Tau Protein Pathologies

The accumulation of Aß in the AD brain precedes intraneural deposition of tau proteins, which has been found to also be a protein hallmark of illness. Toxic aggregates of this protein, which spread via neuron-to-neuron connections throughout the brain, are also typically observed in connection with neurodegenerative events in AD [30,45]. In the diseased brain, axonal, microtubule-associated tau proteins are increasingly phosphorylated by kinases. Hyperphosphorylated tau is able to aggregate to insoluble, filamentous tau structures, which form neurofibrillary tangles (NFTs) in the brain. The hyperphosphorylation of tau is increased by the accumulation of oligomeric Aß, which activate (i) kinases for tau hyperphosphorylation and (ii) inactivate phosphatases for tau dephosphorylation. The hyperphosphorylation of tau leads to tau relocation from axonal microtubules to dendrites [4,46,47]. Tau tangles, fragments, and oligomeric aggregates accumulate in neuronal bodies and synapses, where tau interferes with glutamate receptor trafficking and associated excitation, as well as with neuronal firing. The disruption of synaptic function, accompanied by Aß- and tau-induced loss of axonal myelin, are followed by loss of synapses and neurons. Thereby, neuron cell death is based on apoptosis and neurotransmitter deficiency, and precedes cognitive decline. The hyperphosphorylation of tau has also been related to the decrease in cerebral blood flow (CBF), a further hallmark of AD [45,48]. Overall, these neurodegenerative processes, characterized by the deposition of abnormal tau protein in the brain, are known as tau pathologies or tauopathies. They contribute to AD pathogenesis and are therefore also therapeutic targets in clinical studies for AD [4,45].

### 3.4. Inflammation and Glial Responses

Drug research also focuses on other key players in AD pathogenesis, which include inflammatory processes, associated with glial activity, the production of reactive oxygen species (ROS) and of hydrogen sulfide, vascular and BBB dysfunction, and decline in synapses and neurons [30,35,49]. Particularly, oxidative stress and brain inflammation are early processes in AD pathogenesis. They are closely linked to the generation of Aß and the elimination of neurons and synapses, which are triggered by reactive astrocytes and microglia in the brain [12,50,51].

Under normal conditions, astrocytes and microglia, the brain´s main phagocytic immune cells, are essential to neuronal functioning and health. However, when the brain is injured, infected, or diseased, microglial cells are rapidly activated. They become highly movable, secreting inflammatory proteins, migrating to the affected area, and phagocytosing bacteria, aggregated proteins, cellular debris, and damaged neurons and synapses [52,53]. This intensive reaction of activated microglial cells is generally referred as microgliosis, which includes, in addition to their phagocytosing function, triggering inflammatory processes by releasing proinflammatory proteins. Among these proteins, small cytokine peptides and inflammasome-derived protein complexes are able to stimulate the production, deposition, and spreading of Aß in the brain [54]. Accordingly, cytokines, including some interleukins (ILs) as main triggers of inflammation, induce expression of the IFITM3 (interferon-induced transmembrane protein 3) protein in neurons and astrocytes. IFITM3 protein directly binds to γ-secretase and upregulates its activity, thereby increasing Aß production [55]. In contrast, pharmacological inhibition of the NLRP3 (nucleotide-binding oligomerization domain-like receptor family, pyrin domain containing 3) inflammasome by the small molecule dapansutrile, led to reduced microglia activity and cortical amyloid plaque deposition, accompanied by improved cognitive abilities in AD mouse model [56]. The NLRP3 inflammasome is involved in processing cytokine precursors into active molecules. On the other hand, in an astrocyte-microglia cross-talk observed in AD human and mouse brain, interleukin-3 protein (IL-3) is released by astrocytes and activates the immune response of microglial cells, which then cluster around aggregates of Aß and tau and help to clear them [57]. This corresponds to the known function of microglia, which protects the brain by phagocytosing (engulfing and digesting) and thereby eliminating, e.g., aggregated proteins and unwanted or degenerating synapses [52,53].

In the activation of microglia, the gene triggering receptor expressed on myeloid cells 2 (*TREM2*) has been found to be causally involved [58,59]. This gene encodes a key receptor protein on the surface of microglia in the central nervous system, associated with signaling. TREM2 is thought to mediate repression of inflammatory cytokine production or secretion, and to increase microglial functions in recognizing and removing Aß by phagocytosis. Thus, decrease in CBF and ischemia have been shown to upregulate expression of TREM2 and other phagocytosis-related genes as well as components of astrocytic phagocytosis, suggesting improved ability to remove Aß by glial cells under these conditions [48,58]. Accordingly, loss of *TREM2* gene function led to enhanced amyloid seeding and AD risk [58]. In contrast, acute *TREM2* reduction in AD mouse brain increased microglial phagocytosis, accompanied by slowing Aß plaque deposition [59]. In addition, excessive microglial phagocytosis has been shown to also affect live neurons and synapses, which may contribute to the progressive neuron loss in neurodegeneration [53]. Currently, it is not really clarified which microglial response to cerebral Aß deposition or, generally, to neurodegenerative processes ultimately promotes or inhibits AD pathogenesis [3]. In this respect, studies have shown that microglial cells are able to change their gene expression profiles, dependent on disease progression [52]. This change appears to be associated with a switch in glial activity, from a protective (e.g., by phagocytosing Aß aggregates) to a dysfunctional, neuro-damaging state, which is also known from autoimmune diseases [52].

### 3.5. General Remarks

Undoubtedly, these early disease processes are crucial in the pathogenesis of AD. Therefore, the development of simple biomarker-based tests for the early diagnosis of key events, preferably with blood, is an urgent task of research [60]. Accordingly, in individuals that develop AD, Aß accumulation in the brain has been diagnosed 10–20 years before cognitive impairments are noticeable [61]. Likewise, in the blood serum of persons with hereditary, presympomatic AD, neurofilament light chain (NfL) protein, a fluid biomarker for neuronal cell death, starts increasing 16 years before symptoms appear [62]. NfL is a subunit of neurofilaments, which are components of the axonal cytoskeleton, playing an important role in structural support, transport, and neural transmission. The decay of the axon membrane in the progression of various neurodegenerative proteopathies releases NfL into the ISF and blood stream [62].

## 4. Role of Aß in Triggering Vascular Constriction and CAA in AD

**Figure 1:**
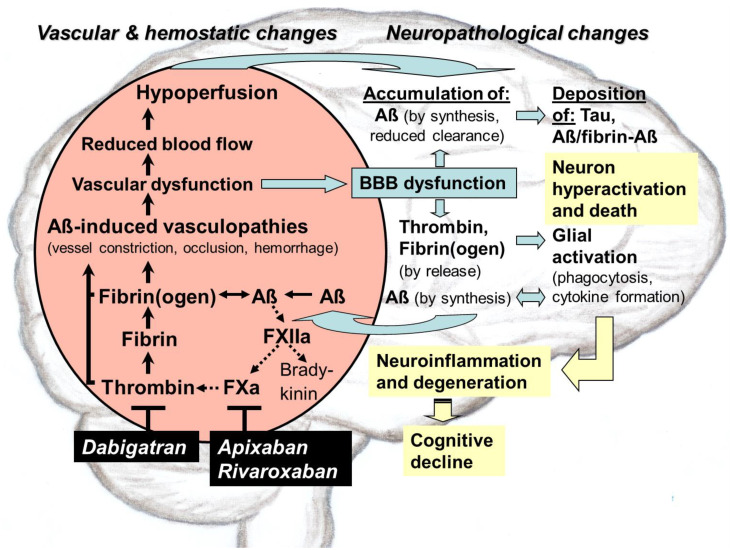
Mechanism of action of direct oral anticoagulants (DOACs) in therapeutic treatment of vascular abnormalities, triggered by thrombin, a key factor in Alzheimer´s disease (AD). In addition to the accumulation of toxic tau and amyloid-ß proteins (Aß) in brain parenchyma, excessive production of thrombin in the blood, leading to fibrin formation; degradation-resistant, Aß-containing fibrin(ogen) clots; and inflammatory milieu are an early and typical hallmark of AD. Particularly in hippocampal and neocortical parenchyma, the release of Aß into the blood triggers the synthesis of thrombin and proinflammatory bradykinin. Aß activate blood coagulation factor XII to generate FXIIa in the plasma contact system. Thrombin, which is produced from prothrombin by the prothrombinase complex via factor Xa (FXa), catalyzes the conversion of fibrinogen to fibrin and induces, together with fibrin(ogen), platelet aggregation, which can lead to vessel occlusion. Deposition of oligomeric Aß and Aß-containing fibrin clots cause vessel constriction and cerebral amyloid angiopathy (CAA). CAA is a major cause in Aß-induced brain vasculopathies and associated lesions, such as vessel occlusion and hemorrhages, leading eventually to vascular and blood–brain-barrier (BBB) dysfunction. As consequences, cerebral blood flow (CBF) and perfusion decrease and supply of brain tissue with oxygen (hypoxia) and nutrients suffer. Concomitantly, Aß increasingly accumulate and aggregates spread in the parenchymal tissue, caused by hypoxia-induced Aß synthesis, as well as by BBB-impaired perivascular Aß clearance. This self-amplifying accumulation of Aß elicits neuronal hyperactivation and synaptic dysfunction and promotes neurotoxic tau pathologies. In addition, BBB dysfunction allows vascular thrombin and fibrin(ogen) to extravasate into the parenchymal tissue and to activate, together with Aß, glial cells, inducing chronic inflammation and further Aß production. In addition, neuronal damage with loss of synapses and neurons is progressing, leading to cognitive decline. DOAC intervention into this vicious circle targets the key mediator thrombin, which can be blocked in its activity by dabigatran or in its production by FXa-inhibitors, such as apixaban, rivaroxaban. Early thrombin inhibition in AD patients could preserve vascular and BBB integrity for full brain perfusion and function. Thereby, vascular-driven neuroinflammation and degeneration and associated cognitive decline could be prevented. Modified from [11].

Cerebrovascular abnormalities, such as vascular lesions (e.g., hemorrhages, tissue injury), vessel occlusion (infarctions), cerebral small-vessel disease (CSVD), impaired vascular function and blood flow, are long-known and very early occurring, typical phenomena in AD. However, only in the last years, Aß-induced cerebrovascular constriction and damage to vessel walls, and their endothelial cells came into focus of therapeutic research [5,12,27,35,36,48,63]. The monolayer of endothelial cells, the endothelium, constitutes the inner lining of the vessel wall in contact with the blood and thus is part of the BBB. BBB is a special structure of brain vasculature that conveys selective and hemodynamically responsive movement of molecules between the blood and the brain [14,63,64]. Pathological deposition of Aß in the vessel wall, which proceeds along with the degeneration of smooth muscle cells, characterizes Aß-type CAA [5,27,34,35,36,37]. Together with early Aß-induced vascular constriction, CAA is a major cause for brain vasculopathies, leading eventually to vascular and BBB dysfunction in AD [27,48]. Moreover, CAA is the most prominent example of crosstalk between vascular and neuronal damage in AD [12,27] (Figure 1). Intriguingly, cerebrovascular amyloid was the starting material for the first isolation and characterization of Aß from AD brain [23].

### 4.1. AD Mouse Models

In order to investigate the mechanism of Aß-caused vascular damage in AD, genetic mouse models are one of the most important research tools, in combination with clinical studies in AD patients [3,65,66]. For preclinical studies, different AD mouse models are available, exhibiting modifications in human AD- and CAA-related risk genes, which are implicated in Aß generation and aggregation, vascular and/or parenchymal Aß deposition, or in cardiovascular risk factors for AD. These models include particularly mice, which are transgenic in human AßPP, Aß, and mutant presenilin of γ-secretase, and mice, which model tau and apolipoprotein E (ApoE) pathology [66].

### 4.2. Occurrence of CAA

Cerebral amyloid angiopathies are commonly observed in the elderly brain and are classified into different types, according to the amyloid protein involved [27,67]. Among these classes, Aß-type cerebral amyloid angiopathy (CAA) is characterized by deposition of congophilic material consisting of Aß in meningeal and small to medium-sized cerebral blood vessels. With a prevalence of 82–98% [67], CAA is most commonly found in patients with sporadic, hereditary, or genetic AD. In the latter case, gene mutations are primarily associated with the Aß pathogenic syndrome [5,12,27,35,36]. CAA is particularly diagnosed in the neocortical and hippocampal brain areas, which correspond to the preferred locations of parenchymal Aß accumulation in AD. In CAA, mainly leptomeningeal and parenchymal small arteries, arterioles, capillaries, and, less frequently, veins are affected in their vascular activity and function through deposition of Aß aggregates in and around vessel walls [5,12,27,35,36,68]. Aß are first observed at the periphery of arterioles, the sites of initial Aß deposition and thus its seeding places [34,69,70]. Depending on CAA severity, Aß accumulation in the vessel wall displays a characteristic pattern with Aß initially deposited in the outer regions of the tunica media to the adventitia. Later, Aß accumulate in all layers of the small arteries and arterioles and can replace vessel wall often totally, except for the endothelial cells. Concomitantly, smooth muscle cells of the tunica media degenerate, associated with massive deposition of Aß fibrils [27,68]. This causes disruption of the vessel wall, showing, e.g., microaneurysms and fibrinoid necrosis. Ultimately, vascular activity and function is lost. Preferentially in capillaries, deposits appear to infiltrate the surrounding parenchymal tissue and accompany dystrophic neurites forming plaque-like structures, known as neuritic Aß plaques [27,68].

### 4.3. Aß in CAA and Brain Parenchyma

Although Aß is the main component of parenchymal neuritic plaques, as well as of vascular deposits in CAA, the length of Aß involved appears to differ between these depositions [27,37]. Parenchymal Aß deposition in AD is composed mainly of Aß42, whereas in the vessel wall of CAA, the shorter subtype Aß40 is the predominant form. According to findings in AD mouse models, the deposition of parenchymal as well as of vascular Aß originates from a common neuronal source [27,37]. This indicates that neuron-derived Aß can migrate to and accumulate in the vasculature far from the site of generation. However, the underlying mechanism has not yet been elucidated in detail [37]. One hypothesis is that Aß42 is withheld in the parenchyma because of its lower solubility and, therefore, its tendency of forming insoluble plaques faster than the shorter Aß isoform. In contrast, the more soluble Aß40 does not aggregate so easily and diffuses more likely along perivascular drainage pathways across BBB into the blood system. In blood vessels of human AD brains, Aß40 accumulates and aggregates on vascular basement membranes, formed by endothelial cells and pericytes [27,37,69]. Thereby, Aß deposits are found first at the periphery of arterioles, alongside of putative ISF drainage routes [34,69,70]. Aß deposition causes vascular damage and dysfunction and impairs perivascular clearance, e.g., of parenchymal waste proteins and Aß, via the distribution and degradation in the blood stream. The perivascular clearance of Aß is also hampered by CAA-related constriction, occlusion, and reduced activity of vessels, as well as by induced thickening of vessel walls. Ultimately, Aß increasingly accumulate in both the brain’s parenchymal and vascular tissue [5,27,34,35,36,37,70,71] (Figure 1).

### 4.4. Brain Vasculopathies and Lesions by Aß-Driven CAA

In the brains of AD patients, CAA-associated vasculopathies lead to the development of hemorrhagic lesions (e.g., lobar intracerebral macrohemorrhage, cortical microhemorrhage, cortical superficial siderosis) and ischemic lesions (e.g., cortical microinfarcts, ischemic changes of the white matter) [27]. Moreover, encephalopathies are associated with CAA-related inflammation [27]. Empirically, CAA is accompanied by multiple or recurrent cerebral microhemorrhages, such as asymptomatic microbleeds and symptomatic, atypical bleeding [67]. The occurrence of new hemorrhages occurs preferentially at the sites of increasing amyloid deposition [27]. In addition, obliterating vascular changes leads to ischemic (micro-)infarcts or lacunes [67], which preferentially affect other cerebral vessels than those that rupture, causing cerebral hemorrhages. However, both events can co-exist at the same time [67].

In the last years, studies in AD mouse models and patients have evidenced that increasing cerebrovascular deposition of Aß is the crucial trigger of CAA. Starting from a self-reinforcing process of Aß accumulation in the vessel walls, the pathogenesis of CAA develops vasculopathies and associated vascular lesions in the brain [27,36,37,68]. These attacks on vessel integrity interfere in vascular and BBB functioning, leading to parenchymal inflammation and neuronal damage, which exacerbate AD pathology and related dementia [27,37,72,73] (Figure 1). Moreover, CAA is also associated with stroke and encephalopathies [27]. In vivo results suggest that probably a mechanism of seeded Aß aggregation, similarly to what occurs in prion diseases, triggers the initiation and progression of CAA and derived vascular defects [27,37,74,75,76]. In these studies, inoculations with Aß-containing extracts from human and mice AD brains, applied intracerebrally [74] or peripherally intraperitoneally [75], elicited sporadic CAA pathology in AD mouse models. Here, in a time- and concentration-dependent manner, Aß-deposition in CAA was strongest in blood vessels of the anterior and entorhinal cortex, with additional deposition in the hippocampus. Concomitantly, massive spreading of Aß deposits was observed into the neighboring brain parenchyma [75]. Likewise, in early onset CAA and brain pathology of patients, who were exposed to Aß from contaminated human sources, such as pituitary gland extracts [69] or cadaveric graft [77], a prion-like mechanism might explain the initiation of both vascular and parenchymal Aß deposition.

These results indicate that CAA-related brain changes obviously not only accompany AD, but causally promote the disease themselves. Accordingly, in the *ApoE-ε4* genotype, the strongest and most prevalent genetic risk factor for late-onset AD, the encoded lipid-transporting ε4 variant of ApoE is deposited together with Aß [78]. This deposition is associated with an increased severity of CAA and parenchymal Aß load [78], as well as with a reduction in glia-mediated Aß clearance [30]. Treatment with the anti-human ApoE antibody HAE-4 in an AD mouse model decreased CAA and parenchymal amyloid plaques and improved cerebrovascular function [78]. According to a recent imaging study in mouse brain, ApoE shuttles astrocyte-derived cholesterol to neuronal membranes, where cholesterol intensifies Aß production by increasing AßPP precursor interaction with ß- and γ-secretase [79]. In addition, in sporadic and hereditary CAA, deposition of tau proteins has also been detected around Aß-accumulating cerebral vessels [37]. However, this vascular tau deposition is thought to be not a prominent feature of CAA [37].

### 4.5. Pathophysiological Impact of Aß on Vascular and BBB Functioning

The CAA-associated vasculopathies and the resulting vascular lesions entail pathophysiological consequences for the Aß-loaded, diseased brain that manifest themselves particularly in the disruption of vascular and BBB functioning [27,48,64,80,81,82] (Figure 1). This dysfunction of the cerebrovascular system is particularly detrimental because it obstructs cerebral blood flow (CBF) and thereby the perfusion of the brain. Full brain perfusion is indispensable to sustain normal tissue metabolism and neuronal functioning. Hypoperfusion restricts the supply of the brain tissue with a variety of vital constituents from the blood, including gas exchange of oxygen and CO2, ions and water, solutes (e.g., glucose and other carbohydrates, fatty acids, amino acids, hormones, vitamins, organic anions and cations, nucleotides), peptides and proteins, as well as cellular components [64,69,80,81,82,83]. In cerebral blood vessels, close correlation has been found between the severity of Aß load and the impairment of vascular function. This dysfunction is expressed in reduced CBF, hypoperfusion (ischemia), and undersupply of the brain, particularly with oxygen (hypoxia) and glucose, a key energy substrate for the tissue [48,76,80,81,84] (Figure 1).

#### 4.5.1. Decrease in CBF Induced by Capillary Constriction and CAA

Reductions in CBF and increases in vascular resistance in the brain are among the earliest pathophysiological events diagnosed in human AD. These events are thought to play a crucial role in driving cognitive impairment [48,81]. Likewise, the importance of blood flow to the brain also emerged from studies that examined physical exercise as therapy for AD [85,86]. Physical exercise stimulated blood flow and thus brain perfusion and nutrient supply. As a result of this therapy, neurodegenerative processes slowed down in people with genetic AD predisposition [85], as well as in an AD mouse model [86]. Usually, chronic reductions in CBF of around 25% are measured in brains of AD patients [70,81,82] as well as in mouse models [48,76], although CBF can even decrease by over 50% in some brain areas [87]. Therefore, ischemia and hypoxia along with reduced brain metabolism are commonly diagnosed in AD [48].

Most of the resistance in the brain´s vascular bed is in capillaries, rather than in arterioles or venules [48]. Consequently, CBF is mechanistically controlled not only by vascular smooth muscle cells around arterioles but also by contractile pericytes, which envelop capillaries from the penetrating arteriole [37]. Therefore, CBF reduction is not only caused by CAA-associated hemorrhagic and ischemic vascular lesions but can also be provoked by the decrease in vessel diameter due to vessel constriction, as observed in human AD brains [48]. Accordingly, CBF in capillaries has recently been found to be reduced by localized capillary constrictions near the pericyte soma [84]. These capillary constrictions are the result of a contraction of pericytes on the outer vessel wall, which is triggered by oligomeric Aß [84]. Indeed, cortical capillary constriction by pericyte contraction is observed early in AD development [48]. The extent of constriction increases rapidly, closely correlated with the severity of capillary Aß deposition, even before CAA leads to pericyte loss in the capillaries [48,84]. Other events that can restrict CBF are the formation of fibrin clots and promoted platelet aggregation in the vessels, as well as the adhesion of neutrophils to the capillary endothelium, which can ultimately lead to vessel occlusion [48,88].

Recently, the Aß-induced mechanism for pericyte contraction and associated capillary constriction in AD has been analyzed in detail in living brain biopsies from patients developing AD and in AD mouse models [84]. The results suggest that Aß oligomers elicit formation of ROS, possibly provided by microglia and pericyte activity. Thereby, ROS seem to trigger a process that induces the release of endothelin-1 (ET) [84]. ET activates contractile, free-calcium-elevating, intracellular endothelin A (ETA) receptors on pericytes. ETA-receptor activation causes the contraction of pericytes and thereby the constriction of capillaries [84]. The increasing capillary constriction and reduction in capillary diameter (vasoconstriction) has been associated with a decrease in CBF, which is commonly diagnosed early in AD [48]. A lasting CBF reduction resulted in chronic hypoperfusion (ischemia) of the affected brain regions and in decreasing levels of oxygen (hypoxia) and glucose in the tissues [84]. However, in this investigation, Aß only induced constriction of the capillaries and did not change vessel diameter of arterioles and venules [84]. In contrast, other studies also indicated the constriction of arterioles and of the middle cerebral artery [48].

Studies have shown that Aß-induced ischemia and hypoxia cause enhanced synthesis of Aß in the brain parenchyma [89,90,91]. The reason is that under these conditions, the cleavage of the AßPP precursor to release Aß is promoted through upregulating ß-secretase 1 (ß-site APP cleaving enzyme 1, BACE1) gene expression and activating γ-secretase [89,90,91]. This feedback loop initiates a self-amplifying process of progressive Aß accumulation in the parenchymal tissue. Concomitantly, tissue metabolism decreases to a level that is expected to fire neuronal inflammation, degeneration, and, ultimately, cognitive decline [48,90,91]. Likewise, in people, who carry the AD gene risk variant *ApoE-*ε4, significant vascular defects have been observed. These include accelerated pericyte loss, BBB disruption, and reduced CBF, correlating with cognitive impairment [92]. Pericytes usually respond very sensitively to ischemia, and along with the increasing deposition of vascular Aß, this could lead to pericyte death and loss of BBB integrity [48]. In addition, Aß-induced ischemia and hypoxia are also able to trigger tau phosphorylation and pathologies, which further promote cognitive decline [48].

#### 4.5.2. BBB Dysfunction and Impaired Aß Clearance

The loss of integrity and function of blood vessels, which vascularize the central nervous system, also concerns the physiological properties of their endothelial cells and thus vessel permeability [63,64]. Within vessels, a continuous endothelial membrane, which has sealed endothelial cell-to-cell contacts by adherens and tight junctions, forms the inner lining of the BBB [64]. A further component contributing to BBB integrity is the vascular basement membrane, a three-dimensional network consisting of proteins, glycoproteins, and proteoglycans, which is formed by enveloping endothelial cells. This structure is sheathed by mural cells (pericytes in capillaries, vascular smooth muscle cells in arterioles and arteries), perivascular immune cells, and surrounding astrocytic endfeet [64]. Capillaries, as the smallest and most common cerebral blood vessels, are major sites of the BBB. The main function of the BBB is to control the transport of, e.g., solutes, ions, peptides, and cells. This transport takes place out of the peripherally circulating blood to the brain ISF and parenchyma. On the other hand, transport also occurs vice versa out of the parenchyma into the blood to clear neurotoxic substances and to ensure a parenchymal milieu, which is appropriate for an immaculate synaptic and neuronal functioning and for its protection [63,64]. CAA-caused vascular changes are an important cause of BBB damage, leading to BBB breakdown and dysfunction, which is also an early hallmark and typical biomarker of AD [64,92]. Accordingly, the *APOE-*ε4 genotype for AD susceptibility has been shown to lead to BBB dysfunction and degeneration of pericytes in brain capillaries, predicting cognitive decline [92]. Generally, BBB breakdown is characterized, e.g., by endothelium degeneration with loss of tight and adherens junctions, increased endothelial bulk flow transcytosis, disturbed BBB transporter systems, pericyte degeneration, and perivascular accumulation of toxic material [64]. BBB breakdown allows the entry of harmful blood constituents into the brain parenchyma, such as neurotoxic molecules, proteins (e.g., thrombin, fibrinogen, fibrin), cells, and microbial pathogens. Concomitantly, the normal transport of blood constituents into parenchyma as well as the regional ISF formation and flow are also affected. As a result, blood-originating cells, debris, water, and electrolytes accumulate in inflated perivascular spaces. This accumulation of diverse and harmful constituents interferes with the normal movement of solutes across parenchymal extracellular spaces and interrupts regional ISF formation and flow [64]. Ultimately, the distribution of solutes is completely disturbed throughout the brain [64]. These detrimental processes are associated with ion dysregulation, swelling of the brain (cerebral edema), and immune and inflammatory responses, leading eventually to neuronal dysfunction, increased intracranial pressure, and neurodegeneration [63,64]. Breakdown of the BBB also impairs perivascular clearance of toxic Aß in the blood. In particular, the mechanisms of Aß transport into the blood via endothelial phosphatidylinositol-binding clathrin assembly protein (PICALM)-mediated transcytosis and low-density lipoprotein receptor-related protein 1 (LRP1) are affected. LRP1 is expressed, e.g., in perivascular astrocytes, pericytes, and, to a lower extent, in capillary endothelial cells. Insufficient perivascular Aß clearance results in an increase in toxic Aß in the parenchyma. Parenchymal Aß accumulation is additionally promoted by a facilitated re-entry of blood-circulating Aß into the tissue. Responsible for this re-entry is the endothelial receptor for advanced glycosylation end products (RAGE), which is present under these conditions in increasing amounts in the micro-vessels [64]. Likewise, CBF reduction hampers Aß clearance and thus amplifies parenchymal Aß accumulation, e.g., by lowering the amount of transporter proteins for Aß relocation and by altering Aß removal by microglia and astrocytes [48]. In addition, both CBF decline and Aß accumulation are known factors that promote tau pathologies [46,64].

#### 4.5.3. Target for Therapeutics

The co-occurrence of Aß depositions in the parenchymal and vascular pathogenesis of AD and the pathophysiological consequences are early mechanisms for triggering cerebrovascular and neurodegenerative changes in the brain, which are thought to lead to cognitive impairment [12,27,48,64,72,73]. One of the earliest pathophysiological events occurring in AD is the reduction in CBF (Figure 1). CBF decline is caused by Aß-induced vessel constriction and CAA-associated vasculopathies and lesions, which include vessel hemorrhage and occlusion, triggered by the deposition of Aß-containing fibrin thrombi [5,48,81,84]. Therefore, Aß-induced cerebrovascular changes and associated CBF decline, leading to extensive consequences on brain functioning, are increasingly coming into research focus today. As integral part of the AD syndrome, these vascular alterations can lead to BBB dysfunction, hypoperfusion and nutrient deficiency in the brain, and accumulations of Aß and inflammatory thrombin and fibrin(ogen) in the parenchymal tissue. In particular, hypoperfusion with concomitant hypoxia cause a dramatic fall in the brain´s metabolism and triggers a self-amplifying process of parenchymal Aß production, deposition, and aggregate spread (Figure 1). This vascular side, contributing to AD pathogenesis, inaugurates new therapeutic approaches for treatment of the disease. Indeed, initial evidence indicates that preserving or restoring normal vascular and hemostatic functioning can maintain cognitive abilities, provided that neurodegenerative damage to synapses and neurons has not yet progressed too far [8,9,11,12,48,93].

## 5. Interaction of Aß with the Plasma Contact System and Its Driven Pathways of Coagulation and Inflammation in AD

A further pathological feature of Aß is that these amyloid peptides interact with distinct components of the plasma contact system and its driven cascades of intrinsic coagulation and inflammatory kallikrein–kinin system in the blood [5,94] (Figure 1). The contact activation system consists of a series of plasma proteins, such as factor XII (FXII), factor XI (FXI), prekallikrein (PK), and high molecular weight kininogen (HK), which are assembled on surfaces of blood cells and vessel walls [16]. The function of the plasma contact system is to initiate the pathways of (i) intrinsic coagulation, leading to thrombin and fibrin production, and (ii) inflammation by the kallikrein–kinin system [5,16]. Interestingly, in AD patients, as well as in AD mouse models, the plasma contact system has been found to be in an activated state. This is already the case in individuals with mild cognitive impairment, who often develop AD [5,94]. When activation of the contact system was inhibited, AD pathology and cognitive impairment were alleviated [13].

Recently, Aß have been shown to possess pro-coagulative activity, leading to activation of FXII in the contact system and thereby promoting the production of inflammatory thrombin and fibrin. Fibrin(ogen) has the potential to aggregate with Aß, forming degradation-resistant deposits of Aß-containing fibrin clots in brain vessels. These clots are thought to be causally related with vascular and BBB dysfunction in AD pathology [13]. Aß-induced FXII activation in the contact system also leads to the production of kallikrein and proinflammatory bradykinin [13]. Kallikrein is thought to favor hemorrhagic conditions, which can enhance the risk of cerebral bleeding. Bradykinin can induce peripheral inflammation and can lead to edema, vasodilation, and increased BBB permeability in AD [13]. BBB leakage allows proteins and cells to migrate from the blood into the brain parenchyma, leading, e.g., to the accumulation of thrombin and fibrinogen in the tissue and to thrombin-mediated formation of Aß-containing fibrin deposits [64].

### 5.1. Aß-Induced Activation of FXII in Contact System and Effects on Pathways Beyond

Recent studies have shown that Aß (Aß42 in particular) can bind and activate blood coagulation factor FXII to generate FXIIa. FXIIa is a crucial factor of the plasma contact system at the intersection between the intrinsic clotting and the inflammatory kallikrein–kinin pathways [13,95,96,97] (Figure 1). FXII is primarily produced in the liver and occurs in the blood. The clotting cascade is triggered by pro-coagulative Aß, when FXIIa activates FXI to FXIa. Subsequently, FXIa initiates a process chain, resulting in the formation of thrombin from prothrombin. Thrombin catalyzes the cleavage of fibrinogen to form fibrin. The inflammatory kallikrein–kinin pathway is initiated when Aß-induced FXIIa activates plasma prekallikrein to generate kallikrein. Kallikrein cleaves HK, which leads to cleaved HK (cHK) and, eventually, to the release of proinflammatory and vasoactive kinin mediators, such as bradykinin [4,13,96,97]. In addition, HK plays an overall role for functioning of both pathways, driven by the contact system, because FXI and PK need to bind HK to be activated by FXIIa.

#### 5.1.1. Indications for Aß Causality

In AD, a role of Aß in triggering FXII activation and the pathways beyond has been suggested by the following findings [13,95,98]. FXII has been found to co-localize with Aß plaques in postmortem AD brain tissue. Moreover, in the plasma of AD patients, an increase in FXIIa levels was accompanied by decreasing levels of FXI, indicating the activation of the intrinsic coagulation system. In addition, activation of the kallikrein–kinin pathway by the contact system has been evidenced by studies showing that the plasma levels of FXIIa, cHK, bradykinin, and of kallikrein activity are elevated in AD patients, correlating with the severity of cognitive decline [13,95,98]. Increased cHK levels were also measured in the CSF of AD patients. Accordingly, intravenous application of Aß42 in wild-type mice has been found to promote HK cleavage and kallikrein activity in plasma [13,95]. Moreover, the activating role of Aß in FXII-driven intrinsic blood clotting and bradykinin synthesis has been supported by biochemical studies [13]. Accordingly, the addition of Aß42 to normal human plasma has been shown to induce the generation of FXIIa, as well as HK cleavage and bradykinin production. These processes were blocked by an anti-HK antibody [13]. Studies using human plasma and purified protein systems also demonstrated that Aß42 stimulate thrombin formation and the subsequent fibrin production [13,95]. This effect appears to be specifically triggered by Aß-mediated FXII activation to FXIIa, because in FXII-deficient human plasma, thrombin formation could not be induced by Aß42 [95]. Moreover, blocking of FXII activation by antibody or FXII antisense oligonucleotide (FXII-ASO) in AD mouse model prevented contact system activation and thrombin production in the plasma [95]. Likewise, treatment of AD mice with FXII-ASO decreased microglia and astrocyte activation, amounts of extravasated fibrin(ogen) in brain parenchyma, and neuronal loss, compared to control AD mice. In addition, cognitive impairment was lowered by FXII-ASO treatment [95,99]. Overall, the results evidenced that Aß42-induced FXII activation in the plasma contact system promotes thrombin and fibrin generation, as well as the synthesis of proinflammatory bradykinin [13,99].

#### 5.1.2. Aß-Induced Accumulation of Thrombin and Fibrin(ogen)

Co-occurring accumulations of Aß, thrombin, fibrinogen, and fibrin have been found in brains of AD mouse models and patients with sporadic and genetic AD, which includes the high-AD-risk *ApoE-ε4* genotype [5,35,100,101,102,103,104,105]. Accordingly, accumulation of the three polypeptide chains of fibrinogen, α, ß, and γ (FGA, FGB, and FGG), have been particularly observed, when Aß were deposited in neuronal tissue and vessels of mouse and human brains during AD development [106]. Thrombin and fibrin(ogen) are important mediators of neuroinflammation. Moreover, fibrin(ogen) can specifically aggregate with Aß [5,100,102,103,104]. These aggregates, consisting of fibrin(ogen) and Aß, deposit along vessel walls as well as in the nervous system parenchyma [5,100,104,105] (Figure 1). Resulting vascular changes damage BBB integrity and function and increase its permeability for plasma proteins, which promotes extravasation of thrombin and fibrinogen from the blood into the brain parenchyma [17,63,73]. Here, fibrinogen leads to the production of fibrin and fibrin deposits, mediated by thrombin and further extravasated tissue factor and procoagulant proteins [17,63,73]. Additional effects of BBB dysfunction are the accumulation of parenchymal Aß due to impaired perivascular clearance, activation of glial cells, and, eventually, neuronal damage [69]. The causes of triggering BBB dysfunction are particularly hemorrhagic and ischemic (occlusive) vascular lesions, which are accompanied by the degeneration of pericytes in the capillary walls, disrupting junctions between adjoining endothelial cells [63].

Along with increasing amounts of fibrin(ogen), Aß, and Aß-containing fibrin clots in the human AD brain [13,95,96], drastic accumulations of thrombin and prothrombin have been detected in cerebral vessels but also in neurons, glial cells, and intraneural tau deposits of the parenchymal tissue [96,107,108]. In AD pathology, both fibrin(ogen) and thrombin can directly trigger a plethora of inflammatory responses in cerebral vessels as well as in parenchymal tissue, particularly due to the activation of microglia and astrocytes and ROS formation [102,105,107,108]. Thrombin is a pleiotropic protease, which is involved in intrinsic coagulation as well as in non-hemostatic processes and can stimulate a multitude of protease-activated receptors (PARs). These thrombin-activated PARs, which are expressed, e.g., on microglia, astrocytes, neurons, endothelial cells, and in blood platelets, contribute to vascular damage, neurotoxicity, and inflammation in AD, particularly through activating glial responses [102]. Via PAR activation, thrombin is also a powerful inducer of endothelial cell activation, as well as platelet aggregation, which is also promoted by fibrin(ogen) [17,102]. Thus, the accumulation of thrombin can lead to the occlusion of vessels, particularly through promoted platelet aggregation and fibrin clot formation. In addition, thrombin has been found to induce epileptic and cognitive dysfunction by disturbing CA3 hippocampal neurons [4,102].

Just like thrombin, fibrin(ogen) can directly activate inflammatory glial responses and ROS generation [17,18,109]. This occurs by binding of the fibrin domain γ377–395 to the microglial CD11b/CD18 integrin surface receptor. In AD mouse models, fibrinogen-mediated microglial activation has been found to be associated with neurodegenerative effects, which include the removal of neuronal dendritic spines and synapses, leading to memory and cognitive deficits [13,17]. Corresponding neurodegenerative effects have also been observed after cerebral injection of fibrinogen, which were even more pronounced in the presence of Aß [13,17]. Genetic disruption or antibody blockage of the fibrin domain, which binds to the microglial CD11b/CD18 receptor, decreased inflammatory reactions, neurodegeneration, and cognitive impairment in AD mouse model [13,109]. In addition to activating microglia, fibrin(ogen) can also trigger the activation of neutrophils, which release toxic ROS and neutrophil extracellular traps (NETs), leading to excessive immune activation and inflammation [110]. An additional inflammatory factor is the fibrinolytic system, converting plasminogen to fibrin-dissolving plasmin [111]. The plasminogen activator system is thought to be involved in promoting brain inflammation, plaque deposition, and BBB dysfunction in AD [111].

#### 5.1.3. Formation of Aß-Containing Fibrin Clots

Both vascular and parenchymal fibrin(ogen) has the potential to synergistically promote Aß-triggered pathology in AD [13,100,103,112]. Accordingly, a prospective observer study revealed that high plasma fibrinogen levels are associated with an increased risk of AD [113]. Moreover, recent research has shown that fibrin(ogen) can specifically aggregate with Aß, leading to the formation of occlusive blood clots in cerebral vessels, associated with vascular and BBB dysfunction, ischemic and hypoxic brain conditions, neuroinflammation, and neuronal death [13,100,101,103,112] (Figure 1). These effects were accompanied with further Aß production and deposition [13,55,89,90,91]. In addition, the disruption of BBB integrity and function led to the release of inflammatory thrombin and fibrin(ogen) from the blood into the brain parenchyma, where fibrin is increasingly deposited, correlated with the severity of neurodegeneration [64,104]. Based on the structure of Aß42, studies have revealed that the central region of Aß binds to the outer D domain of fibrinogen, promoting its oligomerization and formation of Aß-containing fibrin(ogen) clots (fibrin–Aß clots) [100,101,103,112]. The resulting structural abnormalities in the fibrin mesh of these fibrin–Aß clots block their cleavage and degradation by the plasmin fibrinolytic system in the blood. Some mutations in Aß, such as the Dutch- and Iowa-type CAA mutations, led to a 50-fold higher Aß-binding affinity for fibrinogen, which was associated with increasing toxicity and vascular deposition of Aß [13,112]. In AD patients, carrying these Aß mutations of hereditary CAA, an increased fibrin and fibrin–Aß clot deposition was detected in postmortem brain tissue, compared to patients without these mutations [13,112]. It is assumed that the higher Aß-binding affinity for fibrinogen due to these Aß mutations generates structural abnormalities in the fibrin–Aß clots, which further increase their resistance against degradation [13,112]. Usually, Aß-containing fibrin deposits are found around and in cerebral vessels, particularly in neocortical and hippocampal brain areas developing CAA. In these vessels, increasing accumulation of fibrin-Aß clots is associated with vascular and BBB dysfunction and a reduction in CBF [5]. In addition, fibrin–Aß aggregates have also been observed in brain parenchyma, particularly in areas of dystrophic neurites, surrounding Aß oligomers and fibrils plaques [17,100,101,103,104]. In this tissue, progressive accumulation of fibrin–Aß aggregates have been linked with the triggering of inflammatory and neurodegenerative changes and, ultimately, with the death of synapses and neuron cells, leading to cognitive impairment [5].

The results give evidence that the interaction between fibrin(ogen) and Aß to form aggregates is an important factor in triggering cerebrovascular, neurodegenerative, and cognitive disorders in AD. Consequently, blocking this interaction and minimizing this harmful crosstalk could be a further approach for the therapy of AD [13]. Indeed, small molecular inhibitors have been found, which interfere in the interaction between fibrin(ogen) and Aß [5,114]. RU-505 is one of these compounds, which binds directly to Aß42 and prevents Aß-induced structural changes in fibrin clots. Thereby, RU-505 prevented thrombotic and fibrinolytic abnormalities in an AD mouse model [5,114]. Accordingly, the long-term treatment of AD mice with RU-505 reduced amyloid deposition, vessel infarctions, and neuroinflammation in the brain, as well as cognitive impairment [5,114].

### 5.2. Pathological Dimension

The recently detected crosstalk between Aß and FXII, which stimulates production of thrombin, fibrin, fibrin-Aß clots, and bradykinin, as well as associated glial responses, is an important pathological feature, contributing to cerebrovascular and neuronal dysfunction in AD [5,13] (Figure 1). In brain parenchyma, accumulations of thrombin, fibrin(ogen), and Aß generate together a chronic inflammatory milieu [13]. Particularly, glial cells are activated and produce inflammatory cytokines, which elicit continued formation of Aß and spread of their aggregates in the brain. Cerebrovascular dysfunction is caused early by capillary constriction and occlusion, mediated by Aß, fibrin–Aß clots, platelet aggregation, and neutrophil trapping, as well as by developing CAA-related arterial damage, infarctions, and hemorrhage [5,13,48]. The resulting BBB breakdown and dysfunction leads to extravasation of thrombin and fibrin(ogen) from the blood into the brain parenchyma but also, vice versa, to reduced perivascular clearance of parenchymal Aß in the blood stream [64]. All effects together cause the collapse of CBF and brain perfusion, resulting in deficiency of oxygen and nutrients and a drop in the metabolism of the cerebral tissue [5,12,35,36,48]. As consequence, a self-amplifying spiral of increasing accumulation of Aß, thrombin, and fibrin(ogen) is elicited in the parenchyma, which is accompanied by enhanced formation and deposition of fibrin–Aß aggregates and tau pathologies. This further intensifies neuroinflammatory and degenerative processes as well as cerebrovascular damage in AD pathogenesis (Figure 1). Ultimately, loss of synapses and neurons cause memory and cognitive abilities to gradually disappear [5,12,35,36,48].

## 6. Therapeutical Intervention Using Thrombin-Inhibiting Anticoagulants against Dysregulated Intrinsic Coagulation in AD

### 6.1. Rationales for Use: Results from Basic Research

From the theoretical view, there are serious arguments for including the pathway of intrinsic coagulation as a therapeutic target in the strategy to treat AD. In the center of this approach is to address the harmful cerebrovascular changes in AD. These changes lead to the collapse of the vascular and BBB function and of the blood supply for the nervous system parenchyma, causing neuroinflammatory and neurodegenerative responses. An important trigger of this disastrous vascular scenario is Aß-driven, out-of-control production of thrombin in the blood. This thrombin overproduction leads to increasing formation of fibrin and degradation-resistant fibrin–Aß clots, as well as to promoted platelet aggregation and inflammation (Figure 1). Therapeutic intervention into this vicious circle focuses on directly blocking activity or production of thrombin by treatment with anticoagulants. As result, a variety of adverse processes in AD might be decelerated, stopped, or prevented (Figure 1). These include (i) the deposition of fibrin–Aß clots and oligomeric Aß in cerebral vessels, leading to vascular and BBB dysfunction; (ii) reductions in CBF, brain perfusion, and nutrient supply; (iii) the accumulation of extravasated, inflammatory thrombin and fibrin(ogen) in the brain parenchyma; (iv) the accumulation and aggregate spreading of parenchymal Aß by hypoxia- and glia-induced Aß synthesis, as well as by impaired perivascular Aß clearance due to BBB dysfunction; (v) glial activation and associated neuroinflammatory responses; (vi) Aß-induced tau pathologies; (vii) neuronal damage with loss of synapses and neurons, leading to cognitive impairment.

Consequently, a thrombin-inhibiting treatment might offer a therapeutic chance to counteract Aß-induced vasculopathies and dysfunctions in AD, and to prevent hypoperfusion, hypoxia, and nutrient deficits, as well as neuroinflammatory milieus in the brain, elicited by accumulating Aß, thrombin, and fibrin(ogen). As a possible therapeutic success, neuroinflammatory and neurodegenerative alterations, leading to memory and cognitive decline, could be slowed down, stopped, or prevented. However, the therapy of patients should be initiated as early as cognitive deficits by AD are suspected and clinically confirmed. As a precaution, patients with diagnosable CAA should be excluded from the therapy to avoid bleeding risk [9,10,11,13,102].

### 6.2. Rationales for Use: Results from Preclinical Studies

Nearly twenty years ago, the first experimental studies showed that anticoagulant use offers the chance to slow the progression of AD, exemplified by peripheral treatment with heparin-type enoxaparin in AD mouse models [115,116]. These studies demonstrated that the indirect inhibition of thrombin activity and production by enoxaparin can significantly reduce cortical Aß concentration and severity of Aß deposition, as well as amounts of activated astrocytes around Aß plaques [115,116]. Likewise, in in vitro studies, enoxaparin led to lowered Aß neurotoxicity and reduced Aß ability to activate the complement and plasma contact system [115]. In addition, in cultured brain endothelial cells, exposed to hypoxia, as well as in AD mice, direct inhibition of thrombin activity by DOAC-type dabigatran decreased cerebral glial activation [117] and vascular expression of inflammatory proteins and production of ROS [108]. Overall, the results suggested that the inhibition of thrombin could have therapeutic value for treatment of hypoxia, inflammation, and oxidative stress in AD [102,108,118].

However, only recently, the first preclinical study for a detailed proof-of-concept has been carried out by Cortes-Canteli and co-workers [93]. In order to prevent harmful effects of thrombin in AD mouse brains, the authors employed long-term anticoagulation with dabigatran, which directly blocks thrombin activity [93]. The results revealed that dabigatran is able to inhibit the formation of occlusive fibrin thrombi in cerebral vessels, decreases in CBF, brain hypoperfusion, and memory decline [93]. Concomitantly, dabigatran treatment preserved BBB function, proved by pericyte integrity and the absence of AD-related astrogliosis [93]. Likewise, dabigatran treatment significantly decreased amyloid plaque deposition as well as halved the accumulation of surrounding Aß oligomers [93]. Concomitantly, the neuroinflammatory milieu in the brain tissue was reduced, demonstrated by lowered amounts of phagocytic microglia and infiltrated peripheral T cells [93]. In addition, no hemorrhages or incidents of intracerebral bleeding were observed [93]. These findings highly confirm conclusions, drawn from basic research, on possible benefits of a thrombin-inhibiting therapy in AD [9,10,11,13,93,102,118] (Figure 1).

### 6.3. Rationales for Use: Results from Clinical Studies

#### 6.3.1. Historical View on Early Investigations and the Hypothesis of AD Therapy

The first clinical studies on the therapeutic and prophylactic value of anticoagulant medication against dementia, which already included placebo-controlled interventions, extend far back into the 1960s [119,120,121,122]. In these studies of small groups of senile-presenile dementia patients, treatments with the VKAs dicumarol and warfarin resulted in decelerated cognitive decline and reduced morbidity and mortality. Since, at that time, the exact mode of action of this potentially new therapeutic approach against dementia was unclear, these studies were rarely discussed afterwards and fell nearly into oblivion [5,102,118]. In the 1980s, the author has been inspired by observations in his personal environment and by scientific interest to deal with the idea using anticoagulants against AD. Beginning in 2020, this hypothesis has been specified in several review articles, motivated by recent findings on cerebrovascular and hemostatic changes, contributing to neuronal dysfunction in AD [9,10,11,15].

#### 6.3.2. Observer Studies on Patients with Anticoagulant Use due to AF

Over the last 20 years, results from multiple clinical studies and systematic reviews suggest that oral anticoagulant use in AF safeguards against incidence of dementia, as shown in patients without a dementia history before treatment [123,124,125,126,127,128,129,130]. Accordingly, the beneficial effects of oral anticoagulants (DOACs, VKA warfarin) against dementia and cognitive impairment were obtained in a retrospective cohort study (2000–2017), which used UK primary care data from treatment of nearly 85,000 patients [124]. In this study, oral anticoagulant (OAC) use was compared to non-OAC and antiplatelet treatment among AF patients [124]. In addition, results from a retrospective Swedish study (2006–2014) on nearly 450,000 participants indicated a protective effect of DOACs and the VKA warfarin against dementia, whereby the risk of dementia was reduced by 29% [123]. Moreover, a retrospective propensity score-matched cohort study of ca. 5000 elderly individuals from the USA (2010–2014) showed that especially the use of DOACs (apixaban, dabigatran, rivaroxaban) was associated with a reduction in cerebral ischemic events as well as in new-onset dementia, compared with the VKA warfarin [125]. DOAC use also offered superior long-term efficacy and safety in avoiding thromboembolic and hemorrhagic events and death [125]. Likewise, in a retrospective, electronic-health-record-based cohort study of nearly 40,000 individuals at age 40 years or more with new onset AF from the UK (2012–2018), DOAC treatment (apixaban, dabigatran, rivaroxaban) was associated with a reduction in new diagnoses of all-cause dementia and mild cognitive impairment, compared with VKA use (acenocoumarol, phenprocoumon, warfarin) [126]. In addition, recent systematic reviews and meta-analyses of retrospective [127,128] and prospective [129] observational studies and randomized controlled trials concluded that oral anticoagulants can significantly reduce the occurrence of cognitive impairment in patients with AF. Compared with the VKA warfarin, DOACs have shown a superior protective effect on cognition [127]. A recent literature review on the incidence of neuropsychological disorders and dementia in AF patients, treated with dabigatran or warfarin, came to the same result [130]. After the evaluation of a recently terminated prospective clinical study with ca. 100 elderly AF patients, data showed that 24 months of treatment with dabigatran or warfarin was associated with similar efficacies against stroke, cognitive decline, and dementia [131]. Currently, a prospective observational study in ca. 3000 patients with AF is ongoing for up to 84 months to explore the efficacy and safety of rivaroxaban versus standard of care treatment (acetylsalicylic acid in patients with, placebo in patients without vascular disease) in preventing ischemic stroke and neurocognitive impairment [132].

## 7. Clinical Perspective for Anticoagulant Use against AD

The current portfolio of anticoagulants that would be available for a disease-modifying therapy, targeting vascular and associated neuronal changes in AD, consists of drugs which have been used in clinical practice for a long time. Therefore, safety profiles, pharmacokinetics, formulations, doses, and manufacturing processes of these medications are well-known. These preconditions would considerably reduce time and costs for the pharmaceutical development and approval process and, therefore, would have beneficial effects on the cost of their therapeutic use.

### 7.1. Evaluation of Therapeutic Suitability of Available Anticoagulants

For long-term treatment of AD-related vascular dysfunction with anticoagulants, questions for safety, undesirable side effects, and dosage form for daily drug administration play an important role, in addition to the medicinal efficacy.

#### 7.1.1. Parenteral Anticoagulants

The parenteral administration of a drug by injection or infusion directly into the blood stream are less suitable for a permanent therapy from their handling alone. This would be the case when treating parenteral, indirect thrombin-inhibiting heparins (e.g., enoxaparin), heparinoid danaparoid sodium, and fondaparinux or direct thrombin-inhibiting hirudin, bivalirudin, and the synthetic L-arginine derivative argatroban. Heparins, hirudin, and derivatives are usually administered for short-term prophylaxis of thromboembolic events and for therapy of acute venous thrombosis [16]. Undesirable side effects of treatment with heparins are given by abnormally low levels of platelets in the blood (thrombocytopenia) and increased risk of bleeding. In addition, anticoagulation by heparins is unpredictably affected by unspecific plasma protein binding. Furthermore, heparins do not inhibit fibrin-bound thrombin and related thrombus formation [16,133]. In contrast to heparins, hirudin inactivates thrombin bound to clots and does not directly interact with platelets. However, in therapeutic application, bleeding complications have been frequently observed [134]. Intriguingly, in a 20-week pilot study using patients with mild-to-moderate AD symptoms, treatment with hirudin in combination with donepezil alleviated cognitive impairment more effectively than by treatment alone with donepezil as standard therapy [135].

#### 7.1.2. Oral Anticoagulants

With respect of administration and handling, oral treatment with anticoagulants for therapy would certainly be preferable to a parenteral application, if the efficacy and safety profile of the oral drug is also favorable and the patient can take the medication on a consistent basis. For oral administration, anticoagulants are available on the market from VKA-type, such as warfarin, phenprocoumon, as well as from DOAC-type with the direct thrombin inhibitor dabigatran and the direct FXa inhibitors apixaban, betrixaban, edoxaban, rivaroxaban [16]. Dabigatran, rivaroxaban, and apixaban were approved for antithrombotic use already at the beginning of the 2010s years [16]. Approval of edoxaban and, subsequently, betrixaban was in the second half of the decade [16]. The development of the new class of antithrombotic DOACs that directly target to specific factors in intrinsic coagulation was particularly desired due to serious disadvantages of the conventional VKAs [16,20,136].

Pharmacological cons of VKA medication include (i) delayed onset and slow offset of the antithrombotic drug effect; (ii) slow, delayed over hours, antidote action of vitamin K in bleeding situations; and (iii) variability in antithrombotic efficacy due to drug–drug interactions and vitamin K intake by diet [16,20,136,137]. Consequently, close medical supervision of the drug level in the blood of patients is required to ensure efficient anticoagulation as well as to avoid bleeding due to overdose. Nevertheless, risk of bleeding complications increases particularly with increasing age, what is the most important undesirable side effect of VKAs [16]. A further adverse effect of VKAs is the induction of necrosis of skin and subcutaneous tissue, what is a rare, but severe complication, based on protein C deficiency. In addition, VKA treatment can modify the functioning of important vitamin K-dependent proteins in vascular and nervous system, such as growth-arrest-specific gene-6 (Gas6), matrix Gla protein (MGP), and key enzymes of the sphingolipid biosynthetic pathway [16,20,137]. On the other hand, VKAs are not eliminated by the kidneys and, thus, can be used in patients with serious renal impairment [136]. In addition, VKA medication costs are significantly lower than those of DOACs [20,136].

Compared to VKAs, DOACs provide constant therapeutic efficacy and a more favorable safety profile, as well as avoidance of adverse effects from vitamin K deficiency due to their different mechanism of action. In detail, the advantages of DOACs include (i) rapid onset of action, (ii) short half-life, (iii) less drug–drug interactions and no dietary interactions, and (iv) safe antidote strategies in situations of bleeding risk. Therefore, the lower intra- and interindividual variability in the DOAC-effect allows fixed dosing and a predictable anticoagulative response without the need for continuous monitoring of the drug level in patients [9,11,16,20,136,138,139]. Since DOACs, especially dabigatran, are eliminated to a large extent via the kidney, the renal function in patients should be routinely monitored, particularly in elderly persons due to increasing renal impairment and associated co-morbidities [20,136]. In patients with renal impairment, dependent on severity, DOAC use requires dose adjustments or is contraindicated [20,136]. In addition, DOAC-type anticoagulants also hold the risk of bleeding, particularly of serious intracranial hemorrhage [16,20,136].

#### 7.1.3. Risk Assessment of Oral Anticoagulants in Clinical Observer Studies

In a systematic review and meta-analysis of phase III trials for stroke and systemic embolism prevention in patients with AF (2009–2013), DOACs (apixaban, dabigatran, edoxaban, rivaroxaban) showed a more favorable risk–benefit profile compared with warfarin [140]. In addition, reductions in all-cause mortality and systemic embolic events, these agents reduced the risk of hemorrhagic stroke by 51% and the risk of intracranial hemorrhage by 52% [140]. This favorable efficacy and safety profile was consistent with many subgroups and ethnicities including the Asian population [136,140]. Conversely, dose-dependently, the risk of gastrointestinal bleeding was 25% higher with DOACs than with warfarin [140]. Similar results were obtained in a large retrospective observational study of ca. 400,000 AF patients, based on US claims data (2013–2015) [139]. Treatment with DOACs (dabigatran, apixaban, rivaroxaban) was associated with lower rates of stroke and systemic embolism, compared with warfarin. In addition, apixaban and dabigatran showed lower rates of major bleeding, including gastrointestinal bleeding, intracranial hemorrhage, and major bleeding at other key sites, whereas rivaroxaban had a higher rate of major bleeding, compared with warfarin [139]. This is in accordance with results from a new-user retrospective cohort study of patients with AF and dementia (2011–2017), comparing DOAC treatment versus warfarin [141]. DOAC-treated patients, who were older and had more comorbidities than the warfarin-treated individuals, showed similar prevention of thromboembolic events, compared to warfarin, but a reduced risk of intracranial bleeding [141]. However, the risk of gastrointestinal bleeding was increased in the DOAC treatment [141]. Lower rates of major bleeding and mortality were also observed in a randomized, double-blind trial of ca. 18,000 AF patients, when apixaban use was compared with warfarin [142]. Apixaban was also superior to warfarin in preventing stroke or systemic embolism [142]. In addition, efficacy and safety of treatment with dabigatran versus warfarin was comparatively investigated in a retrospective observer study of ca. 130,000 elderly AF patients (2010–2012) by FDA [143]. Dabigatran was associated with reduced risk of ischemic stroke, intracranial hemorrhage, and death, but with an increased risk of major gastrointestinal bleeding, compared with warfarin. However, most importantly, dabigatran reduced the incidence rate for dangerous intracranial hemorrhage by 66%, from 9.6 per 1000 person-years in the case of warfarin to 3.3 in dabigatran use [143]. Dabigatran also showed a more favorable safety behavior in comparison with rivaroxaban [144]. In this retrospective new-user cohort study of ca. 120,000 elderly AF patients (2011–2014), rivaroxaban use was associated with an increase in intracranial and extracranial hemorrhage, such as gastrointestinal bleeding, compared to dabigatran [144]. A favorable safety behavior of dabigatran was also evident from investigations in mouse models of AD and CAA [145,146]. In these studies, dabigatran use was not associated with an increase in intracerebral hemorrhage and incidence of acute micro-bleeding [145,146].

### 7.2. DOAC-Type Anticoagulants for In-Depth Clinical Investigation

Altogether, the wide range of clinical observer studies, conducted in the last 15 years particularly in elderly individuals with AF, demonstrate that DOAC-type anticoagulants exhibit a predictable therapeutic effect in preventing stroke and systemic embolic events. In addition, DOACs show a safety profile that more than halves the risk of dangerous intracranial hemorrhage in elderly people compared to VKAs [136,140,143]. These properties, along with their pharmacological advantages, give DOACs a clear preference over VKAs, particularly when anticoagulants are administered to elderly people, who are more vulnerable individuals. In addition, gastrointestinal bleedings, which are more likely to be promoted by DOACs compared to VKAs [136,140,141], can be treated better and stopped immediately after occurrence by effective antidote strategies. These strategies have been successfully developed for various DOACs in the recent years [16]. In the case of dabigatran, the specific antidote idarucizumab (Praxbind^®^) was introduced in 2016 [147]. This antibody binds to dabigatran with high affinity and leads within minutes to a rapid cancellation of the anticoagulative effect, e.g., in emergency operations or in situations of uncontrollable bleeding [16,147]. In the case of the FXa inhibitors apixaban and rivaroxaban, andexanet alfa (AndexXa^®^) was recently approved as a fast-acting antidote [16,148]. Andexanet alfa is a recombinantly modified, human FXa molecule, which itself has no effect on blood clotting. It acts as a kind of decoy protein that binds the FXa-inhibitors and thus restores blood clotting [16,148]. Generally, availability of an efficient and fast acting reversal agent should be a prerequisite for a long-term anticoagulative treatment. This is especially the case in elderly and comorbid AD patients, showing inherent bleeding risk due to fragile blood vessels [68].

#### 7.2.1. Direct Thrombin Inhibitor Dabigatran

One suitable candidate for a clinical study in AD is the oral pro-drug dabigatran etexilate, which is, after absorption, converted by unspecific esterases from plasma, erythrocytes, and liver cells to the active antithrombotic agent dabigatran [11,16,20,138]. Among the available DOACs, the particular feature of dabigatran is that the drug directly binds, competitively and reversibly, to existing thrombin and blocks both fibrin-bound and free thrombin activity [138] (Figure 1). Consequently, dabigatran prevents thrombin-mediated formation of fibrin and of associated degradation-resistant fibrin–Aß clots, as well as the accumulation of inflammatory fibrin in brain vessels and parenchymal tissue. In addition, dabigatran blocks the inflammatory activity of soluble thrombin, leading, e.g., to promoted platelet aggregation, enhancement of Aß production, and neurotoxic brain changes [102,138]. Dabigatran also exhibits anti-inflammatory potential, which has been demonstrated in a fibrosis mouse model [149]. Overall, dabigatran use could offer a chance to combat AD-related cerebrovascular and BBB dysfunction and to prevent brain hypoperfusion and nutritional deficiency and associated neuronal and cognitive decline (Figure 1).

#### 7.2.2. FXa-Inhibitors Apixaban and Rivaroxaban

Among FXa-inhibiting DOACs, apixaban and rivaroxaban are particularly interesting candidates for AD treatment in a clinical study. This conclusion is especially based on their medicinal efficacy, availability of a broad clinical data base on bleeding risk, and presence of a fast-acting antidote [9,10,16,148]. Apixaban and rivaroxaban block the production of coagulative and inflammatory thrombin through direct and reversible inhibition of free and prothrombinase-bound FXa [16,20] (Figure 1). This results in decreased formation of fibrin and fibrin–Aß clots. Concomitantly, the inhibition of thrombin production reduces thrombin-mediated platelet aggregation and inflammation in the cerebrovascular system. Consequently, pathological thrombin effects on vascular and BBB function and brain perfusion and nutrient supply can be counteracted. In addition, associated neuroinflammatory and neurodegenerative processes can be prevented. However, contrarily to the direct thrombin inhibitor dabigatran, FXa-inhibitors cannot abolish the activity of already existing molecules of thrombin and their inflammatory impact [16,20]. On the other hand, these small amounts of residual thrombin can allow FXa inhibitors to uphold some functioning of hemostasis, which may be associated with a lower risk of bleeding, compared to direct thrombin inhibition [16].

### 7.3. Concluding Remarks

As outlined in Section 6 and Section 7, particularly thrombin-inhibiting DOACs offer a great potential for treating of AD. This conclusion is based on results from (i) mechanistic studies on the role of Aß, thrombin, and fibrin(ogen) in provoking vascular and neuronal dysfunction [5,17,35,102], (ii) preclinical investigations in AD mouse model [93,108,115,116,117], and (iii) clinical observational studies in patients with AF [123,124,125,126,127,128,129,130,131]. Therefore, clinical investigation of this new therapeutic approach in patients with diagnosed AD in early-stage is highly recommended [9,10,11,35,93,102]. However, no clinical placebo-based study with anticoagulants in a substantial cohort of AD patients had been conducted up to now. In this connection, a first double-blind pilot trial for evaluating disease-modifying effects of the thrombin inhibitor dabigatran in AD patients (50–85 years of age, 24 months treatment) was announced in 2018; however, it has not yet initiated [150].

After considering the pros and cons of the available DOACs, which come from basic and preclinical research, clinical observer studies, data bases on bleeding risk, and availability of antidote, the thrombin inhibitor dabigatran and the FXa-inhibitors apixaban and rivaroxaban are on the list of preferential drugs for a clinical study in AD. Both types of thrombin-inhibiting DOACs should be able to effectively combat effects of an out-of-control production of thrombin in AD, which result in excessive fibrin–Aß clot formation and deposition, the promotion of platelet aggregation, and the generation of thrombin- and fibrin(ogen)-induced inflammatory milieus [9,10,11] (Figure 1).

## 8. Conception for a Clinical Intervention Study with DOAC Treatment

For an early and preventive treatment of vasculopathies and their impact in AD, a prospective, placebo-controlled clinical study is proposed that should ideally be performed by neurologists in close cooperation with cardiologists. Ultimately, the goal is to investigate whether thrombin-inhibiting DOACs, such as dabigatran, apixaban, and rivaroxaban, could be a therapeutic option to counteract the early occurrence of vascular dysfunction and related neuronal and cognitive disorder in AD.

### 8.1. Bleeding Risk and CAA Development

An urgent prerequisite for the study is that participants showing hereditary or spontaneous AD are screened carefully with regard to their bleeding risk, e.g., according to the criteria of clinical HAS-BLED-Score and imaging methods [11,16,151]. An exclusion criterion for participation would be, when CAA-associated intracerebral bleeding is detected, e.g., by MRI imaging sequences with high sensitivity for bleeding, according to the criteria for diagnosing CAA [151]. Specific biomarkers, which could characterize procoagulant and CAA state, would also be very helpful for recognizing and better pre-selecting patients with a risk of serious cerebral bleeding for a clinical trial [48]. If those biomarkers, which are currently under investigation, e.g., for thrombus detection by molecular imaging [152], are available, they could be used not only for early CAA diagnosis, but also for accompaniment of a therapy. This is important, since the manifestation of brain microbleeds often lags behind dementia development and, therefore, patients could be endangered through a longer therapeutic or prophylactic AD treatment with DOAC. In addition, special care should also be taken for patients at risk of gastrointestinal bleeding, such as those with inflammatory bowel disease, diverticulosis, and angiodysplasia, because DOAC treatment can increase their bleeding risk [20,136,140,141].

### 8.2. Methods for AD Diagnosis

As outlined before, DOAC treatment should start as early as possible to maintain a significant hub over untreated patients in a study, as well as to largely avoid unwanted bleeding events. However, in the early stages, diagnosis of AD and differentiation from other dementias can be difficult and is often ambiguous. This makes a sophisticated and in-depth diagnosis of AD and its pathogenesis essential.

#### 8.2.1. Interviews and Neuropsychological Testing

For the selection of suitable participants for a study, interviews on their anamnesis, subjective condition, and perceived cognitive symptoms are the starting point for a first and preliminary AD diagnosis. This is followed by objective and sensitive neuropsychological testing of the cognitive impairment, which also accompanies therapy monitoring throughout a study.

#### 8.2.2. EEG, MRI, and PET Imaging

The technological platform for AD diagnosis makes use of electroencephalographic (EEG) recordings for assessment of neuronal abnormalities. However, preferentially, magnetic resonance imaging (MRI) and position emission tomography (PET), which show higher spatial resolution, are applied [4]. MRI and PET imaging techniques are particularly useful for detecting the known brain biomarkers for AD. These biomarkers include cerebral deposition of extracellular Aß and intraneuronal neurofibrillary tangles (NFTs), rich in hyperphosphorylated tau protein (pTau), as well as cortical and hippocampal atrophy and neuron loss, reductions in glucose metabolism, decrease in CBF, and hemorrhagic events [4,68,80,153,154]. However, amyloid-PET as well as tau-PET only quantify fibrillar deposits in the brain, while soluble, neurotoxic oligomers are not recorded [30]. In addition, MRI and particularly PET imaging are rather expensive diagnostic tools.

#### 8.2.3. Invasive CSF Analysis

Invasive methods, such as cerebrospinal-fluid (CSF) analyses, also allow the examination of patients for biomarkers of amyloid, tau, and neurodegeneration (ATN biomarkers). Methods for determining fluid CSF biomarkers include, e.g., immunoprecipitation-mass spectrometry of Aß42/40 concentration ratio, immunoassays for fragments of Aß (Aß42), total tau and pTau species, and axonal protein NfL as a neurodegenerative marker [60,62,154,155], as well as liquid chromatography-mass spectrometry for proteome analysis of glial activity [156]. Although in clinical practice techniques of neuropsychology, MRI and PET imaging, and CSF diagnostics are increasingly refined, they typically only give a clear diagnosis for AD in the case of already distinct disease symptoms. These methods reveal a less clear picture in the very early stages of the disease. However, the prerequisite for a therapeutic study with anticoagulants is an early diagnosis of AD, before significant symptoms have already emerged. In addition, diagnosis should also allow clear differentiation of AD from other dementias.

#### 8.2.4. Diagnosis by Blood-Based Tests and Intestinal Microbiome Analysis

ATN biomarker diagnosis using blood-based tests is a new option for screening and therapy monitoring of patients, which combines early AD diagnosis with manageable effort [2,60,155]. By its progress in developing sensitive blood-based biomarkers for AD, blood phenotyping can be an attractive and cost-saving alternative or supplement to MRI/PET scans and invasive CSF analyses in the near future [60,155]. Currently, the available ATN biomarkers already allow, e.g., biochemical analysis of Aß fragments, Aß42/40 ratio, tau fragments, NfL, and glial fibrillary acidic protein (GFAP) [62,154,155,157], as well as the determination of Aß-misfolding using immuno-infrared-sensor technology in automated platform [157] and analysis of microRNA signature linked to neural homeostasis [158]. In a recent study using a cohort of participants with AD diagnosis and without dementia followed over 17 years, four plasma biomarkers were measured at baseline [157]. Aß misfolding showed high disease prediction accuracy of AD and, among the concentration markers, GFAP exhibited the best performance, followed by NfL and pTau181. A combination of Aß misfolding and GFAP increased the accuracy in discriminating between AD and controls and showed a strong ability to predict AD risk [157].

For a clinical study on DOAC therapy, an appropriate selection of available blood ATN biomarkers should be complemented by analysis of coagulation biomarkers, especially thrombin and fibrin(ogen). A new, non-invasive method that can be possibly also used for AD diagnosis in future derives from changes in the composition of the intestinal microbiome, which result in a specific AD signature. This method has been shown to discriminate amyloid-positive AD patients from cognitively healthy, elderly individuals [159].

### 8.3. Clinical Perspective of DOACs

#### 8.3.1. Limitations and Qualification Scenarios for Investigation

In an ethically justifiable intervention study with DOAC treatment, possible benefits and, above all, the personal risk of bleeding have to be evaluated extensively for each participant and discussed in detail before consent. Furthermore, it is crucial for the therapeutic success and for the safety of the application that subjects are at the very beginning of cognitive AD symptoms and CAA pathogenesis. This is also the lesson concluded from recent clinical studies on Aß-targeting therapies [2,4,7]. It would be ideal if individuals who are cognitive symptomless but highly likely to begin developing symptoms on a measurable timescale could be treated [2]. Therefore, sensitive and simple tools are preferred, which can unequivocally diagnose changes in typical AD biomarkers years before the onset of cognitive symptoms. Analytically, a combination of blood-based ATN biomarker tests and MRI imaging techniques for AD and CAA diagnosis could initiate the appropriate start of treatment, when the first symptoms of cognitive impairment be feared or are perceived. Possibly in the future, biomarkers on procoagulant and CAA state will also be available and complement the procedure. In pre-symptomatic individuals with high familiar or genetic risk of early onset AD and CAA, sensitive blood-based tests could be used to detect first signs indicative of the impending brain disease, which could initiate a prophylactical treatment with DOAC. Most important is to treat and prevent this terrible disease before it begins [2]. For analytic monitoring of the course of a therapy, in addition to continuous blood-based ATN biomarker testing, brain imaging and CSF analyses should be used at regular intervals. This is necessary to confirm the initial and ongoing blood-based biomarker test results and to supervising therapeutic approach. In this context, it is also particularly important to calculate the costs/benefits for the planned analyses at an early stage.

#### 8.3.2. Drug Options for Therapeutic Approach

In order to verify the therapeutic value for targeting thrombin as a pathological key mediator of vascular and neuronal dysfunction in AD, dabigatran as well as apixaban and rivaroxaban could be reasonable DOAC candidates for a clinical pilot study. This conclusion is particularly based on (i) long experience in antithrombotic use and efficacy, (ii) extensive retrospective and prospective clinical observer studies on patients with AF, (iii) broad clinical databases on bleeding risk, and (vi) the presence of a fast-acting antidote for managing emergency operations or situations of uncontrollable bleeding [9,11,16,20,136,138,140,143,147,148].

When taking a look in the distance future, an interesting approach could also be to search for multimodal-acting agents, which directly combat both thrombin-related cerebrovascular dysfunction and neuronal changes in AD [160]. However, this would most likely mean the development and approval of a completely new active ingredient, which causes high costs and takes a long time.

#### 8.3.3. Future Direction towards DOAC Repositioning for AD

A prospective, placebo-controlled clinical intervention study showing that DOAC use is able to slow, stop, or prevent the incidence of cerebrovascular, neuronal, and cognitive symptoms might be a trigger for therapeutic translation of the drug towards approval for AD. This effort is facilitated by the fact that dabigatran, apixaban, and rivaroxaban are long-prescribed anticoagulants with favorable safety and antidote profile, used in millions of patients. However, the prerequisite is that clinical institutes with neuropathological and cardiological competence, supported by pharmaceutical industry and funding associations, get off the ground of the study [9,10,11]. The arguments given in this review shall be an impetus for this intention.

#### 8.3.4. Other Brain Amyloidosis with Associated Vascular Dysfunction

Current research also concerns the question, whether vascular dysfunction, leading to reduced CBF and hypoperfusion in AD, also plays a role in other brain amyloidosis, such as Parkinson´s disease (PD) and Huntington´s disease. In PD, the formation of intraneuronal inclusions of misfolded α-synuclein proteins (called Lewy bodies) and prion-like spread of the toxic α-synuclein, is a key pathological hallmark that is associated with progressive neuronal death but also with vascular alterations and decreased CBF [26,48,161]. Accordingly, the overexpression of human α-synuclein in PD mouse model caused pathological changes in the cerebrovascular system, which were accompanied by BBB leakage with extravasation of fibrinogen into brain parenchyma and pericyte activation [161]. In addition, transcriptomic analysis along the arteriovenous axis of patients with Huntington´s disease revealed transcriptional changes in cell types, which are critical for the maintenance of vascular and BBB integrity [162]. Overall, these findings allow one to speculate that DOACs might be suitable not only for treating cerebrovascular amyloidosis in AD but also in other neurodegenerative diseases. However, a prerequisite is in-depth research of amyloidosis-induced cerebrovascular and hemostatic changes and associated neuronal dysfunction [17,163].

## Abbreviations

Aß	Amyloid-ß proteins
AßPP	Amyloid-ß protein precursor
AD	Alzheimer´s disease
AF	Atrial fibrillation
ApoE	Apolipoprotein E
ATN	Amyloid, tau, and neurodegeneration
BBB	Blood–brain barrier
CAA	Aß-type cerebral amyloid angiopathy
CBF	Cerebral blood flow
cHK	Cleaved HK
CSF	Cerebrospinal fluid
DOAC	Direct oral anticoagulant
EEG	Electroencephalography
FDA	U.S. Food and Drug Administration
FSH	Follicle-stimulating hormone
FXI	Blood coagulation factor XI
FXII	Blood coagulation factor XII
FXa	Activated factor X
FXIIa	Activated factor XII
ISF	Interstitial fluid
HK	High molecular weight kininogen
MRI	Magnetic resonance imaging
NfL	Neurofilament light chain protein
PAR	Protease-activated receptor
PET	Position emission tomography
PD	Parkinson´s disease
PK	Prekallikrein
pTau	Hyperphosphorylated tau protein
ROS	Reactive oxygen species
t-PA	Tissue plasminogen activator
TREM2	Triggering receptor expressed on myeloid cells 2
VKA	Vitamin K antagonist
